# Understanding Barriers and Facilitators to Healthy Eating and Active Living in Rural Communities

**DOI:** 10.1155/2014/146502

**Published:** 2014-12-11

**Authors:** Rebecca Seguin, Leah Connor, Miriam Nelson, Andrea LaCroix, Galen Eldridge

**Affiliations:** ^1^Division of Nutritional Sciences, Cornell University, Ithaca, NY 14853, USA; ^2^Friedman School of Nutrition Science and Policy, Tufts University, Boston, MA 02111, USA; ^3^University of California, San Diego, CA 92093, USA; ^4^Montana State University, Bozeman, MT 59717, USA

## Abstract

*Objective*. Studies demonstrate that people's food and physical activity (PA) environments influence behavior, yet research examining this in rural communities is limited. *Methods*. Focus groups of 8–15 women were conducted in rural communities in seven US states. Questions were designed to identify factors within residents' food and PA environments they felt helped or hindered them from eating healthfully and being physically active. *Results*. Participants were aged 30–84 years; mean (SD) = 61 (14) (*N* = 95). On average, communities had fewer than 5,000 residents. Limited time, social norms, and distances from or lack of exercise facilities were common PA barriers. Facilitators for PA included social support, dog walking, and availability of affordable facilities. Healthy eating barriers included the perception that healthy foods were too expensive; calorically dense large portion sizes served at family meals; and frequency of eating foods away from home, which were perceived as generally unhealthy. Healthy eating supports included culture/value around local food gathering (e.g., hunting and gardening) and preservation (e.g., canning and smoking). Friends and family were frequently identified as key influencers of eating and PA behavior. *Conclusions*. Targeting both social and built environment factors, particularly those unique to rural locales, may enhance support for healthy eating and PA behavior change interventions.

## 1. Introduction

The consequences of obesity are well known. They include risk of developing chronic diseases such as cardiovascular disease, hypertension, diabetes, and several types of cancer [1]. The prevalence of obesity is higher in adults who live in rural areas compared to those who reside in urban areas [2, 3], and rural adults are less likely to meet physical activity guidelines than urban residents [1].

The influence of the built environment on physical activity and healthy eating behavior is an important issue, with the Centers for Disease Control, Institute of Medicine, and World Health Organization all prioritizing changes in built environment as one of the top recommendations for increasing physical activity [4–6]. Many aspects of built environment barriers to physical activity and healthy eating are common to both rural and urban areas, including cost of accessible food and recreation, access to healthy foods, and the “walkability” and “bikeability” of communities [7–10]. However, rural residents may face additional obstacles, such as travel distances from recreational facilities and lack of facilities themselves [11, 12]. Research also suggests that people in rural areas who aim to exercise outside (as a remedy for the lack of or distance from exercise facilities) may face barriers such as weather extremes and safety issues such as busy roads, lack of sidewalks and lighting on streets, loose dogs, and the presence of hunters during hunting season [13–22]. Likewise, access to healthy, affordable foods may be a problem in rural areas: in small grocery, convenience, or village stores there may be a lack of high-quality, healthy options or high costs. Stores and restaurants with more nutritious selections may be a long distance away [16, 23–25].

Despite these unique considerations, research examining barriers and facilitators of physical activity and healthy eating in rural communities is limited. Much of the emphasis has been on youth [26–39] and areas with large population groups [9, 12]. Additionally, research in rural areas has often included participants from only one or two communities and/or limited geographic scope [14–16, 21, 40]. The present study aimed to better identify and understand common rural barriers to healthy eating and active living in a geographically diverse, rural sample.

## 2. Methods

**Figure 1:**
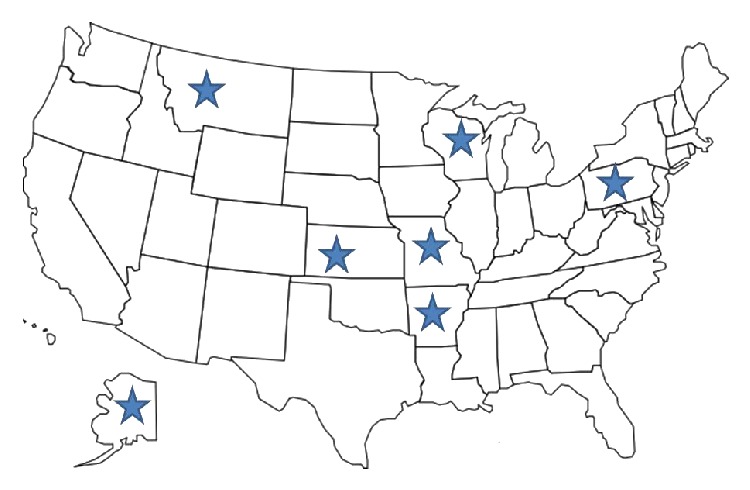
Geographic representation of study sample.

The research team traveled to seven rural communities in seven states in the USA as part of a larger project, called the StrongWomen Change Club project (manuscript currently in review). StrongWomen Change Clubs (SWCC) were developed through an academic-community research partnership guided by community-based participatory research principles. Local leaders, all cooperative extension educators with whom our research team has been working for the past decade, each recruited 10–15 residents to undertake a project to improve some aspect of the nutrition or physical activity environment. Most SWCC participants had limited (or no) experience in civic engagement and/or policy work. At 6- and 12-month after implementation, the research team conducted key informant interviews with each SWCC leader to capture their perceptions of program process, benchmark achievement, and self-efficacy. The first step in implementing the project was to better understand barriers and facilitators to healthy eating and active living among residents in those rural townships, defined as a population of fewer than 15,000 people. Focus groups were conducted with community members in each of the following states: Alaska, Arkansas, Kansas, Missouri, Montana, Pennsylvania, and Wisconsin (Figure 1).

Seven focus groups (one in each location, with SWCC participants) were conducted (by RAS or MEN), lasting approximately 45–60 minutes per focus group. Researchers asked open-ended questions about factors that influence physical activity and healthy eating among residents. They asked residents to think about both the barriers and facilitators that contribute to their exercise and eating habits in their community. In addition to participating in the focus groups, participants were asked to complete a brief, self-report questionnaire that contained demographic and health behavior questions derived from standard inventories (e.g., Behavioral Risk Factor Surveillance System (BRFSS)). All materials and procedures were approved by the Tufts University Institutional Review Board. All study activities were conducted with the understanding and consent of the study subjects.

### 2.1. Analysis

Qualitative data analysis was conducted by LMC and RAS between May and October 2013. Transcript files were entered and coded using NVivo (QSR International, version 9.0, 2011), led by LMC with a subset of double coding and agreement checks between RAS and LMC. Manifest content analysis was used to analyze the data, employing the following process: verbatim transcription; transcription review by multiple coauthors; development of and connection between codes and themes (based upon the main focus group questions and the addition of emergent subthemes); and data interpretation and review by the research team in an iterative process until agreement was reached. Quantitative (survey) data were analyzed by RAS using SPSS version 21.

## 3. Results

The main focus group questions informed the initial development of the coding structure with the original questions serving as the main themes: (1) barriers to physical activity; (2) facilitators to physical activity; (3) barriers to healthy eating; and (4) facilitators to healthy eating. Reoccurring subthemes were identified through an iterative process and confirmed for agreement. Themes and subthemes are displayed in Table 1.

Demographic characteristics of participants are displayed in Table 2. Focus groups contained 8–18 female participants (*N* = 95) per focus group. The average (SD) age of the 95 women was 59.3 years (14.0). The majority of the women were white (94.7%), married (68.1%), and educated beyond high school (92.6%). Focus group participants (82.4%) described the area they lived in as very rural or mostly rural. The average population size of the seven towns was 4,747 residents. Demographic characteristics of participants were compared to the overall population using census data for each town (American Community Survey 2010).

Survey responses related to physical activity and nutrition are displayed in Table 3. Survey responses indicated that more than half of the women were overweight or obese (57.9%) based on self-report height and weight (shown as body mass index (BMI)). Women self-reported their health status as excellent (20.0%), very good (49.5%), or good (28.4%), based upon a 5-point Likert scale. The majority of participants (63.2%) considered themselves active, defined as “generally active daily and exercise two or more times per week.” Exercising was usually performed in a combination of settings including inside at home, outdoors, and/or inside at a facility (71.0%). Most participants (51.6%) engaged in physical activity in a combination of ways including alone, with one other person, and/or in a group or class. Thirty-eight percent of participants reported excellent access to physical activity facilities and 22.3% reported excellent access to healthy foods within their neighborhood or community.

### 3.1. Perceived Barriers to Physical Activity

Barriers to physical activity included lack of time and competing priorities; activities that promote sedentary behavior; social norms and stigma; and built environment barriers.

*Lack of Time due to Busy Work Schedules and Competing Priorities*. Time emerged as a major barrier to engaging in physical activity. Very busy schedules often made exercising at the end of a work day seem impossible.

*…people work constantly to make ends meet.*

*Whatever I do has to be at the end of the day. And some days, at the end of the day, all I want to do is go home…ya know, because my day has been busy.*

Several women describe a tradeoff between engaging in physical activity and forfeiting some of their already limited sleep time when exercising in the morning is their “best option.”

*Because if I do not (exercise in the morning) I can think of a million excuses.*

*Yeah, cause being active for me means less sleep, which means getting up in the morning and doing it and that is hard to give up.*

*…we are not talking giving up from like eight hours of sleep or ten hours of sleep, we are getting like five hours of sleep, so you are giving up from that, so now you are getting four hours of sleep so that you can exercise.*

*Competition with Activities That Promote Sedentary Time*. Women discuss how sedentary activities that they enjoy like watching television and reading consume their time and make it difficult to engage in activity outside of the household.

*…the television is on all of the time, there is always this stimulation we do not necessarily need with the television in our bedroom and umm, just kind of being addicted to…having this tube speak at you or speak around you all of the time, it keeps us kind of in the house.*

*…I think that screen time, computer time, any kind of sedentary time has become a big part of our lives.*

*Social Norms and Stigma*. Using active transport, such as bicycling to work, had a negative stigma associated with it. One woman suggests that cycling to work must be caused from losing a driver's license.

*So this is my question, if I get a bicycle and start riding it to work, are you all going to think that I lost my driver's license? I go by this house and this man evidentially, bless his heart, lost his driver's license for several years, because I have driven that route before and I have seen him riding (his bicycle) for over a year.*

*Built Environment Barriers and Geographic Isolation*. Furthermore, cycling and walking in some communities were considered unsafe due to fast traffic, busy roads, and poor street safety features. Rural residents often have to travel long distances to access exercise facilities due to the separation between commercial areas and residential housing.

*…it has been several months now, but we did have a high school kid (driving a car) hit a woman on a bicycle.*

*…that is one of the problems too, getting a route from my house to work that is safe.*

*I do not want her (my daughter) to walk by herself because she has to cross a four lane highway and I am not ready for that to happen yet.*

*On the other hand, if you live a long ways out, are you gonna get up, come in town, exercise, get all hot and sweaty, drive back home, shower, and get yourself back to work, I am not going to do that.*

*When we moved here forty years ago, the post office was in the middle of town, the shopping A&P was, you could walk. Those days I walked, pulled a little wagon with the kids in it, cannot do that anymore.*

### 3.2. Perceived Facilitators to Physical Activity

Facilitators to physical activity included building physical activity into daily routines; social support; and affordable and accessible fitness venues.

*Building Physical Activity into Daily Routines*. Building physical activity into daily routines was reported by many of the women. Women reported that they received a lot of their physical activity through yard work, gardening, and household chores. Some women consciously modified their normal habits to actively incorporate more physical activity into their daily lives.

*…I try to get up every commercial and go do something…you would be amazed at how much you can get done if you get up and do it during those three to five minute segments. You can almost clean your entire house in a few hours.*

*I volunteer at the hospital and I have tried to walk up and down the stairs as much as I can.*

*…park a couple of blocks down the street when you are on Main Street from where you want to go and walk the whole Main Street.*

*In the summer, I swim on my lunch hour. And when I started doing that, I told my bosses that that was what I was going to do, and I would come back with wet hair and it wasn't an issue, it wasn't an issue for them at all, which was good.*

*Social Support*. Many people report that their pets keep them active. In addition, living in a small community allows interaction among people who have common interests which further promotes physical activity.

*I walked before I had a dog, but the dog made me walk even more. And then I met more people when I was walking the dog and then we kind of all walked together.*

*And I had some old guy who is retired now who stopped once; he used to ride his bicycle around. He stopped once…(A friend) and I walked every morning, and he stopped to tell us that he was going to be gone on vacation, so we wouldn't worry if we did not go by him.*

*…you have to have that encouragement from somebody else.*

*Affordable and Accessible Fitness Venues*. Access to affordable physical activity venues contributes to the promotion of physical activity within communities.

*I did think about our community as a whole and how much healthier our community has gotten since they put the fitness club in, since they have offered these physical activities.*

*And they open it (fitness center) up…you do not have to be a member; you can just come once and pay three dollars.*

*…they are making it affordable and a lot of people are taking…their spinning classes were all full from what I understand.*

*(A friend's) insurance pays for her to go to fitness.*

### 3.3. Perceived Barriers to Healthy Eating

Barriers to healthy eating included lack of time and competing priorities; cost of healthy food; adjusting habits to favor a healthier diet; and geographic isolation.

*Lack of Time and Competing Priorities*. The time required to shop and prepare food was identified as a major barrier for people who already struggled with busy family and work schedules. Commute time for people who reside in rural communities compounded the issue. Eating out or relying on quick microwave meals often took the place of eating a home-cooked meal.

*…all of the handy dandy little things that you can buy and have in the cupboard and then your kid can just pop in the microwave and they got their own dinner in just a minute, ya know? I think that that is a barrier for people…the convenience.*

*I think that our restaurants are a big challenge, ya know, because of my work schedule, we do eat out once or twice a week sometimes.*

*I have worked a lot with people with kids that are very active with school activities, tons of after school activities. These parents run constantly. Family time for these families is in the car on the way to a ball game eating something from a fast food place.*

*And part of it in the rural areas is if you have kids active in sports, you are not driving to facilities in the same town, you are driving thirty miles, an hour away for these ball games. You get off work, you get in the car, and when you get home that night, you are not putting something in the crockpot for the next night. And so ya know, all of the driving and distance we have.*

*Cost of Healthy Food*. The cost of fresh food was identified as a barrier to eating healthy, especially among low-income members of the community. Purchasing produce at the local farmers' market was costly and the Supplemental Nutrition Assistance Program (SNAP) benefits were not accepted at most markets. Furthermore, offering a healthier substitute is a great way to promote healthy eating; however, when the healthier option is more costly, it can be seen as a barrier.

*And what is adequate access for some of us does not mean that it is for everyone. Some of us have more resources than a lot of the community.*

*But like our farmers' market does not do EBT (electronic benefit transfer) cards and things like that.*

*The farmers' market here is more expensive then supermarket food sometimes.*

*…the soft drink comes free (with the meal), if I want to get them (kids) milk, that costs me extra.*

*Difficulty Adjusting Habits to Favor a Healthier Diet*. Adjusting long-term habits of cooking and eating was difficult for many women. Women reported cooking large, high calorie meals for their husbands that farmed or engaged in physically demanding labor. As men got older and duties on the farm subsided, adjusting for the reduction in calorie requirements was difficult.

*…some of us here were raised on a farm and when four men have been out working all day and so even though my husband does not farm fulltime now, I think I have to cook…and so that mindset is really hard to get through, and I can blame it on my husband, but it is really my mindset.*

*That was the way with me, we lived on a farm for years and we had mashed potatoes, chicken, gravy, sometimes twice a day, and then ya know, as you get older, you do not feel like you need all that, or you should not.*

*Food Environment Barriers and Geographic Isolation*. Access to healthy food options is often limited by the long distances that residents from rural communities must travel to get to these sources.

*I wouldn't say that we have excellent access, I would say we have adequate access. There are a lot of things that you cannot get in (small city) that you can get in (larger city) which is forty minutes away.*

*But again, they (farmers' markets) are not everywhere, you have to drive to them. That's just our ruralness, I understand that too, but…*

*Difficulty Avoiding Unhealthy Food at Community Venues and Gatherings*. Making the decision to select a healthy option is not always easy. Furthermore, it can be difficult to avoid unhealthy food at community events or through organizations that offer food as rewards or incentives.

*A lot of the churches cook and people go and eat at the church. And so you eat what they offer because you do not have to cook it. But it is lasagna and things of that type, chicken pot pies.*

*The bank is a barrier for me. Cause there are fresh cookies on the inside…and I have three small children who know that.*

### 3.4. Perceived Facilitators to Healthy Eating

Facilitators to healthy eating common to focus group participants included ability to grow and produce food and access to farmers' markets and farm shares.

*Ability to Grow and Produce Food*. Several women reported growing and producing their own food. They not only had access to fresh food, but were able to control the production process as well.

*I raised one hundred chickens this summer, but I pumped only what I wanted into them.*

*I have a 105 (square) foot asparagus patch and blueberries.*

*Access to Farmers' Markets and Farm Shares*. Participants felt that having access to local farmers' markets and having the opportunity to join a community farm share have supported healthy eating.

*You sign up (for the CSA-Community Supported Agriculture) in the beginning of the year and you pay for kind of a share of the farm…and they have a wonderful newsletter, which for me is useful for recipes on how to use this stuff.*

*I think we have accessibility too to fresh meats, meats that are locally grown.*

*We also have some farmers doing options now where you get combined things in bulk. They were doing those two or three times a week during the summer.*

## 4. Discussion

Much of the research examining barriers and facilitators to active living and healthy eating among residents in rural communities has only focused on one geographic location [15, 16, 22, 40]. This study aimed to gather perspectives from a broad geographic range—from Alaska to Pennsylvania and in between.

Interestingly, there were findings that are similar to what has been reported in studies not focused on rural locales—particularly lack of time and competing priorities to exercising and eating healthfully.

Our study also supports and expands upon existing research related to barriers to physical activity and healthy eating specific to rural communities such as busy roads and fast traffic, poor access to fitness facilities, expensive costs of healthy food, difficulty adjusting eating habits like consuming large portions, lack of variety and quality of food at local stores, and difficulty avoiding unhealthy food at community events and gatherings [14–16, 22, 24, 40]. Primary facilitators to active living and healthy eating in this study also confirm prior findings, highlighting factors such as accessible and affordable fitness facilities, social support, and access to fresh and local food [15, 24, 40].

Participant and community characteristics from prior studies differed from our sample of participants with respect to gender [24, 40], education [24, 40], geographic location [14–16, 22, 24, 40], and race [15, 40]. This suggests that similar barriers and facilitators exist around healthy eating and active living in rural communities and across a range of demographic characteristics.

A major theme that was discussed in four of the seven focus groups was the attempt to build physical activity into daily routines. In some instances, this meant that participants proactively sought ways to increase their activity such as waking up early to work out, parking further away from destinations, taking the stairs when an elevator is an option, and engaging in activity on lunch breaks. In other cases, physical activity was naturally a part of daily routines like housework, laundry, gardening, and walking the dog.

Several themes identified may be specific to rural communities. A unique finding that emerged from the focus groups is the stigma associated with being physically active in some of these small rural communities. If a person was routinely seen riding a bicycle or walking to work it might be assumed that he/she had lost his/her driver's license. This finding may be specific to rural communities where active transport is not common and residents in the community can easily identify one another.

Changing dietary habits is difficult and the reduction of perceived barriers, increased overall health concern, social modeling, and self-efficacy may be necessary to take action and maintain change [41]. Our findings suggest that making dietary changes can be difficult, especially when women are accustomed to preparing large portions of high-caloric food for men who have physically demanding jobs such as farming and field labor. As these men age and transition away from these physically demanding occupations, adjusting cooking practices can be a difficult challenge unique to rural and farming households.

As documented in other studies, rural residents may have improved access to healthy foods due to the availability of fresh and local produce offered through farmers' markets and roadside stands [24, 40]. Our participants discussed in detail how they produced their own food. Joining farm shares and acquiring local meats and produce were commonly reported as well. Although access to healthy foods may be limited in traditional food stores, other alternative options to acquiring fresh produce and meats are available and may be more common among rural communities.

Many of the subthemes identified in our study were interconnected across other subthemes and main themes illustrated in this project. For example, living in a rural community may require long distance travel to access food or physical activity venues which may put a strain on budgets and time. Our participants identified the cost of healthy food and lack of time as barriers to engaging in physical activity and healthy eating. The interconnection among these themes may amplify the barriers that rural residents face. In prior research, Wilcox et al. found that many of the facilitators identified in focus groups were opposite to the barriers and vice versa [15]. Some of our results indicated a similar pattern. For example, access to farmers' markets and farm shares was identified as a facilitator to eating healthfully, while long distances from food outlets was discussed as a barrier. Social support was viewed as a positive contributor to engaging in physical activity while social norms and stigma acted as a barrier to engaging in active transport such as cycling.

The main limitation to this study is the inclusion of primarily white, educated women, which hinders the generalizability of our findings. However, the women were generally representative of the communities visited. Also, we identified several common barriers and facilitators in this work compared to past research that suggests that key themes are stable across demographic groups. Another limitation is the lack of urban comparison groups. Because of this, we are not able to conclude that the themes that emerged are specifically associated with rural communities. Lastly, social desirability bias may have occurred if focus group participants felt that they could not express personal barriers or if they overreported or exaggerated positive physical activity or healthy eating behaviors.

Overall, this study fills an important gap in knowledge related to barriers and facilitators to physical activity and healthy eating in rural communities. Future intervention strategies should target the identified factors and place specific emphasis on the overlapping themes that emerged from our study and past studies. In addition, addressing stigma related active transport, adjusting cooking practices and habits, and increasing awareness of local food options could be of particular interest when working with rural communities.

## Figures and Tables

**Table 1:** Themes and subthemes identified through focus groups.

Barriers to physical activity	
Lack of time and competing priorities	
Competition with activities that promote sedentary behavior	
Social norms and stigma	
Built environment barriers and geographic isolation	
Facilitators to physical activity	
Building physical activity into daily routines	
Social support	
Affordable and accessible fitness venues	
Barriers to healthy eating	
Lack of time and competing priorities	
Cost of healthy food	
Adjusting habits to favor a healthier diet	
Food environment barriers and geographic isolation	
Difficulty avoiding unhealthy food at community venues or	
gatherings	
Facilitators to healthy eating	
Ability to grow and produce food	
Access to farmers' markets and farm shares	

**Table 2:** Demographic characteristics of focus group participants (*N* = 95).

Age (mean, SD)	59.3	14.0

	*N*	%

Married	64	68.1
Race		
White	89	94.7
African American	1	1.1
American Indian/Alaskan Native	4	4.3
Education		
High school	7	7.4
Some college/technical school	19	20.0
Associate's degree	8	8.4
Bachelor's degree	26	27.4
Master's degree or higher	30	31.6
Graduate or professional	5	5.3
Rural classification^a^		
Very rural	24	26.4
Mostly rural	51	56.0
Suburban	4	4.4
Somewhat urban	12	13.2

^a^ *N* = 91.

**Table 3:** Survey responses of focus group participants (*N* = 95).

BMI		
Underweight, ≤18.5	1	1.1
Normal, 18.5–24.9	34	35.8
Overweight, 25–29.9	34	35.8
Obese, ≥30	21	22.1
Missing	5	5.3
Health status		
Excellent	19	20.0
Very good	47	49.5
Good	27	28.4
Fair	1	1.1
Poor	1	1.1
Diet		
Very healthy	10	10.5
Mostly healthy	55	57.9
Sometimes healthy, sometimes unhealthy	30	31.6
Somewhat unhealthy	0	0.0
Unhealthy	0	0.0
Physical activity		
Very active	17	17.9
Active	60	63.2
Sometimes active	17	17.9
Mostly inactive	1	1.1
Inactive	0	0.0
Physical activity habits^a^		
Mostly alone	17	18.3
Mostly with one other person	7	7.5
Mostly in group or class	21	22.6
Combination	48	51.6
Physical activity venues^a^		
Mostly inside at home	5	5.4
Mostly outdoors	12	12.9
Mostly inside at facility	10	10.8
Combination	66	71.0
Excellent access to physical activity facilities^b^	36	38.3
Excellent access to healthy foods^b^	21	22.3

^a^ *N* = 93.

^b^ *N* = 94.
